# Effect of *Enterococcus faecalis* Biofilm on Corrosion Kinetics in Titanium Grade 4 Alloys with Different Surface Treatments

**DOI:** 10.3390/ma16134532

**Published:** 2023-06-22

**Authors:** Jadison Junio Conforte, Cecília Alves Sousa, Ana Claudia Rodrigues da Silva, Allan Victor Ribeiro, Cristiane Duque, Wirley Gonçalves Assunção

**Affiliations:** 1 Department of Dental Materials and Prosthodontic, Araçatuba School of Dentistry, São Paulo State University (UNESP), Sao Paulo 16015-050, Brazil; ceciliasousa_alves@hotmail.com (C.A.S.); wirley@foa.unesp.br (W.G.A.); 2 Department of Preventive and Restorative Dentistry, Araçatuba School of Dentistry, São Paulo State University (UNESP), Sao Paulo 16015-050, Brazil; claudia.silva@unesp.br (A.C.R.d.S.); cduque@foa.unesp.br (C.D.); 3 Birigui Campus, Federal Institute of São Paulo, Sao Paulo 16201-407, Brazil; allanvrb@ifsp.edu.br

**Keywords:** bone-integrated endo-osseous dental implantation, titanium, corrosion, biofilms

## Abstract

*E. faecalis* has been associated with bacteremia, sepsis, and bacterial endocarditis and peri-implantitis. This microorganism can remain in the alveolus even after extraction of the root remnant. This study aimed to evaluate the corrosion on different surfaces of commercially pure titanium (Ti) grade 4 (Ticp-G4) as a function of the bacterial biofilm effect of *Enterococcus faecalis*. A total of 57 discs were randomly divided according to their surface finish (n = 19). For microbiological analysis (n = 9), the discs were placed in 12-well plates containing *E. faecalis* culture and incubated at 37 °C for 7 days. The results show that for the intergroup analysis, considering the “electrolyte” factor, there was a difference between the groups. There was greater biofilm formation for the D.A.Zir group, with greater electrochemical exchange for Biofilm, and the presence of biofilm favored greater electrochemical exchange with the medium.

## 1. Introduction

Titanium (Ti) is a material with high tensile strength, good hardness, good tribological property, low specific weight, and exceptional biocompatibility, with use in its pure form (98% Ti) or in conjunction with other metals and as a constituent of orthopedic and dental implants [1,2,3]. Titanium dental implants have their surfaces modified for better osseointegration, either by sandblasting or acid action, and have clinical superiority compared to their smoother counterparts [4]. These indicate that superior bone healing can be achieved in implants with modified surfaces [5,6].

Although these surfaces favor osseointegration, topographical variations in their structure increase the initial colonization of microorganisms. As a result, biofilm formation becomes faster—manifesting inflammation and peri-implant infections [7,8,9,10,11].

Ti is a highly unstable material. When it comes into contact with air or water, a thin layer of titanium oxide (TiO_2_) is formed. This prevents the discharge of ions into the body [1,2]. When exposed to adverse conditions in the oral cavity (e.g., change in pH; presence of fluorine; thermal, chemical, and mechanical changes; presence of biofilm; and saliva), this oxide layer can be degraded, and ions are released to both the internal and external environments. In cases of corrosion of the implanted surface, alterations of these structures culminate with the release of inflammatory mediators at the implanted site [12,13].

With the widespread use of Ti implants and prosthetic crowns in the oral cavity, there is an accumulation of biofilms on these materials. These facilitate periodontal inflammation and microbial colonization [14,15,16]. Although the implants are sterile, bacterial colonization start to occur in the oral cavity (where the implants were placed) 30 min after being in contact with oral tissues. The bacterial composition of the biofilm found in these sites is close to the microbiota of neighboring teeth or similar to the bacteria already present in the oral microflora of an individual [14,16,17]. After the establishment of the biofilm on the surface of dental implants, there is inflammation and subsequent destruction of bone tissues adjacent to the installed implants. This is similar to the condition observed in natural teeth during periodontal inflammation. Hence, when the earlier situation evolves to periodontal infections, it can result in loss of dental implants [16].

It is assumed that corrosion of metallic materials in the oral cavity can be caused by the metabolic activity of organic acids from living microorganisms. This generates an acidic environment that leads to the formation of local electrochemical cells. The reasons for this formation can be attributed to a potential difference in the concentrations of chemicals such as oxygen and direct or indirect ion transfer reactions with substances released by microorganisms [18,19].

Previous studies have indicated that immersion in metabolites, released by bacteria and microorganisms, causes titanium to corrode [20,21]. Furthermore, electrochemical measurements performed in these studies indicate that the electrical charge on the titanium surface was modified to form phosphate-buffered hydrogen peroxide. This was followed by corrosion occurring with the formation of local electrochemical cells [19,20,21,22]. However, these studies were carried out without microbial biofilm formation, exclusively evaluating the corrosion process in Ti in the presence of these substances of microbial cell metabolism.

Among all the microorganisms in the oral microbiota, *Enterococcus faecalis* (*E. faecalis*) was chosen for this experimental study. This is because *E. Faecalis* is a Gram-positive bacterium, survives starvation, has a pH of 11.5 and a facultative anaerobe, releases toxic metabolites, and is capable of colonizing the gastrointestinal, genitourinary, and oral tracts [23,24,25,26]. In recent years, *E. faecalis* has been associated with bacteremia, sepsis, and bacterial endocarditis—which is of great clinical importance [27,28,29,30]. It has also been linked to causing endodontic infections and peri-implantitis. This microorganism colonizes necrotic root canals in apical periodontitis, the primary cause of apical periodontitis in teeth with infected root canals, which can remain in the socket even after the extraction of the root remnant. Consequently, this leads to failure of rehabilitation with implants [26,27,30]. In multirooted teeth with periradicular lesions, the prevalence of *E. faecalis* at the site after extractions is approximately 78% [27,28]. Therefore, this study aims to analyze the adhesion of bacterial biofilms of *E. faecalis* on different surfaces of titanium grade 4 (Ticp-G4) alloys along with the application of an electrochemical test 7 days after the formation of biofilms on the surfaces.

## 2. Materials and Methods

### 2.1. Specimen Preparation

A total of 57 Ticp-G4 discs, with a diameter of 8 mm and thickness of 2 mm, that were supplied by DSP Biomedical^®^ (Campo Largo, Paraná, Brazil) were used in this study.

Initially, all discs were embedded in colorless self-curing acrylic resin (JET^®^, Classic Dental Articles, Campo Limpo Paulista, São Paulo, Brazil), polished, and cleaned using standardized methods of metallography that was described in a previous study [30,31].

The discs were washed with deionized water and 70% propanol. They remained in an ultrasonic vat for 10 min to remove the metallic particles from the wear of the metallography process so that there are no impurities in the specimens. The discs were then dried using hot air at 45 °C (THORTON ultrasonic washer, model USC 2850, Tecnal Laboratory Equipment Ltd., Piracicaba, SP, Brazil).

**Figure 1:**
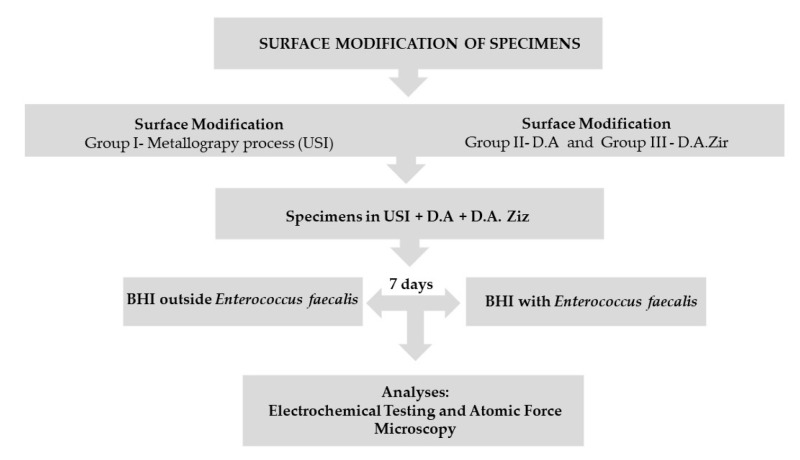
Flowchart.

After the metallography process, the Ticp discs were removed from the acrylic resin using a minicut drill (Wilcos, Petrópolis, Rio de Janeiro, Brazil), which was attached to a straight piece and then washed and dried, as per the method mentioned previously. Subsequently, they were randomly divided into three groups (that is, n = 19 for each group) according to the surfaces to be evaluated, listed as follows: (a) machined surface (Group I—Universal Scanning Interferometry (USI)), which represents the control group; (b) surface texturing by acid etching/etching (Group II—double acid (DA)) (nitric, sulfuric, and hydrochloric acid); and (c) texturing by acid etching/etching, followed by blasting of zirconia particles (Group III—D.A.Zir) (nitric, sulfuric, and hydrochloric acid and sandblasting of zirconia particles). These were carried out in accordance with the standards established by the company in the implantology sector responsible for surface treatment (Figure 1).

Through atomic force microscopy (Flex AFM—Nano Surf) the surface of each group was analyzed, quantifying the area of each specimen to allow obtaining the parameter of the area in order to allow the performance of the electrochemical test. Atomic force microscopy allowed us to observe changes in the topography of the specimens before and after the tests. The USI, D.A. and D.A.Zir groups had a real area of 0.5024 cm^2^, 0.5343 cm^2^, and 0.683 cm^2^, respectively.

### 2.2. Bacterial Strains and Growth Conditions

For the biofilm adhesion assay, nine specimens from each group were selected and evaluated for the corresponding bacterial cell count—measured in colony-farming units per mL (CFU/mL). For the electrochemical assay, 10 specimens from each group were used. Five of the selected specimens were immersed in brain–heart infusion (BHI) medium (without microbial growth for biofilm formation on the discs), representing the control. The other five specimens were selected after going through the week-long incubation period for the adhesion of the *E. faecalis* biofilm. This was necessary to assess the impact of the biofilm during this period on the different titanium surfaces.

All discs were chemically sterilized with ethylene oxide. *E. faecalis* (ATCC 29212) was used for the biofilm formation in the specimens. The bacterial strains were seeded on plates containing m-Enterococcus agar medium (Difco Laboratories^®^, São Paulo, SP, Brazil) and incubated under anaerobic conditions in a 5% CO_2_ anaerobic oven at 37 °C for 24 h. After bacterial growth, these colonies were cultivated in BHI broth medium under the same conditions as the strain reactivation to obtain the microorganism growth curve—determined by optical density (OD) values. After reaching the logarithmic (log) growth phase (phase of greatest cell multiplication), which was 0.5, the culture was adjusted by diluting in BHI broth to a consistency of 1.5 × 10^5^ cells/mL [32].

Sterile specimens were placed on the bottom of 24-well microtiter plates, and 1 mL of the *E. faecalis* culture was added to each well. Bacterial suspensions and sterile BHI culture medium were incubated in separate empty wells for the positive and negative controls, respectively. The plates were incubated at 37 °C for 168 h (7 days) in an anaerobic incubator. Every 2 days, 0.5 mL of each bacterial suspension well was interchanged for 0.5 mL of the BHI culture medium to avoid saturation of the medium and to have microbial viability.

After 7 days, the non-adherent cells were removed from the discs by washing with 1 mL of sterile saline solution to ensure that the non-adherent bacteria would come off as well [33]. Then, the discs were inserted into Falcon-type tubes containing 1 mL of saline solution and shaken for 10 min using ultrasound (USC 700; UNIQUE Ultrasonic Cleaner, São Paulo, SP, Brazil) at 50 kHz (150 W). Similarly, this was repeated in a tube-type shaker Vortex (Vórtex Biomixer QL–901, Curitiba, PR, Brazil) for 30 s to promote the detachment of adhered cells and to obtain the suspension of the biofilm; this was achieved by first collecting 10 μL of the solution and then transferring it to the saline solution in a microtube with 90 μL of this solution. Subsequently, seven serial dilutions of the collected solution was performed.

However, the part a microtube with 90 μL of this solution is still unclear. Each dilution was plated with two drops (25 μL) from each microtube in a petri dish with BHI agar culture medium, in triplicate, and kept at 37 °C under anaerobic conditions for 24 h. The colonies observed on the plates were counted using a manual colony counter, and the number of CFU/mL was thus obtained for each biofilm sample.

### 2.3. Electrochemical Testing

For the electrochemical test, 10 specimens from each group were used (as described in Section 3.2). Further, 10 mL of sterile BHI culture medium was used as the control electrolyte, and 10 mL of the BHI and *E. faecalis* culture medium (1.5 × 10^5^ cells/mL) was used as the test electrolyte. The microbial solution electrolyte only permitted active bacteria to interact with the biofilm on the surface of the Ticp-Gr4 discs. This set-up was maintained at 37 °C, which is close to that of the oral cavity.

Electrochemical tests were performed on the specimens following a previously described protocol [31,34]. All measurements were obtained using a standardized three-cell electrode method in accordance with the instructions (G61 and G31-72) detailed by the American Society for Testing of Materials (ASTM) [31].

Initially, the Ticp-Gr4 discs were subjected to a cathode potential (−0.9 V vs. SCE) to ensure standardization of the oxide layer on their surface. Notably, the cathode potential of 1.8 V vs. SCE for 24 h can cause an eradication of planktonic and biofilm-associated bacteria below detectable levels [35], while the cathode potential of 1.5 V vs. SCE for 4 h does not [36]. That is, the adopted 0.9 V vs. SCE cathodic potential did not affect cell viability. The open circuit potential (OCP) was monitored for 3600 s to assess the potential of the material against the solution and its potential to stabilize the system.

The corrosion parameters were obtained using potentiodynamic polarization curves. The electrochemical impedance spectroscopy (EIE) results were used to model the corrosion process and shed light on the properties of the oxide film that formed on the surface of the Ti discs using the most appropriate electrical circuit. Specifically, Gamry Echem analyst software version 6.2 (Gamry Instruments, Warminster, PA, USA) was used to simulate the EIE data (oxide layer capacitance (double layer) and polarization resistance—Rp).

### 2.4. Atomic Force Microscopy

A sample of the specimens (n = 1) from each group was analyzed under an atomic force microscope (FlexAFM—NanoSurf AG, Liestal, Switzerland, with C3000 controller with ADC and 24-bit DAC), before (Baseline and Biofilm) and after corrosion testing (Baseline and Biofilm). The analysis provided the area of each specimen, as mentioned in Section 3.1, indicating the real area (considering the peaks and valleys) captured by the alteration of the surface of the specimens. The measurements were performed in tapping mode (Dynamic Force) at a rate of 0.5–1 Hz. The tips used were Tap190Al-G-10 (BudgetSensors, Sofia, Bulgaria) with a radius of less than 10 nm. The cantilever used has a force constant of 48 N/m with a resonance frequency of 190 kHz (nominal values provided by the manufacturer). The scanned area was 50 µm × 50 µm with a resolution of 512 × 512 pixels. The images and topographic parameters were analyzed with the aid of the Gwyddion software (version 2.55, Czech Metrology Institute, Brno, Czech Republic). The background of the slopes was fixed and, subsequently, standardized three-dimensional (3D) images were obtained on the *z*-axis scale to enable a visual comparative analysis between the groups.

### 2.5. Results Analyses

GraphPad Prism 7 was used to analyze the results. For the statistical test of the microbiological assay, ANOVA was adopted, and Tukey’s post-test was applied at a significance level of 5% (*p* < 0.05).

For the electrochemical test, a 2-factor ANOVA test (surface and electrolyte) was performed. Each parameter of the corrosion data (i.e., Ecorr, Icorr, Ipass, Cdl, and Rp) was submitted to the non-parametric statistical test of the Shapiro–Wilk and Tukey post-tests for multiple comparisons (*p* < 0.05).

## 3. Results

### 3.1. Microbiological Test

**Figure 2:**
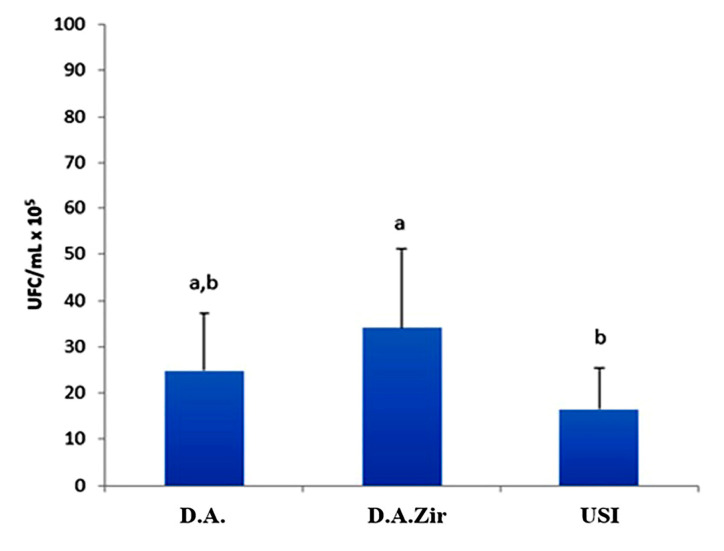
Comparative graph between D.A., D.A.Zir, and Universal Scanning Interferometry (USI) groups considering the average of CFU/mL × 10^5^, according to ANOVA and Tukey tests (*p* < 0.05; different lowercase letters indicate the statistical difference).

Regarding the analysis of CFU/mL, the D.A.Zir group had the highest mean CFU/mL (34 CFU/mL × 10^5^), showing a statistically significant difference when compared to the USI group (*p* = 0.034). However, when compared to group D.A., the difference was not statistically significant (*p* = 0.393), despite that the mean value of CFU/mL was higher (24 CFU/mL × 10^5^) (Figure 2).

It was also observed that the USI group had a smaller amount of biofilm formed (16 CFU/mL × 10^5^) on the surface when compared to the other groups (D.A.Zir = 34 CFU/mL × 10^5^; O.D. = 24 CFU/mL × 10^5^). However, there is no statistically significant difference compared to group A. A. (*p* = 0.416).

### 3.2. Electrochemical Testing

#### 3.2.1. Open Circuit Potential (OCP)

**Figure 3:**
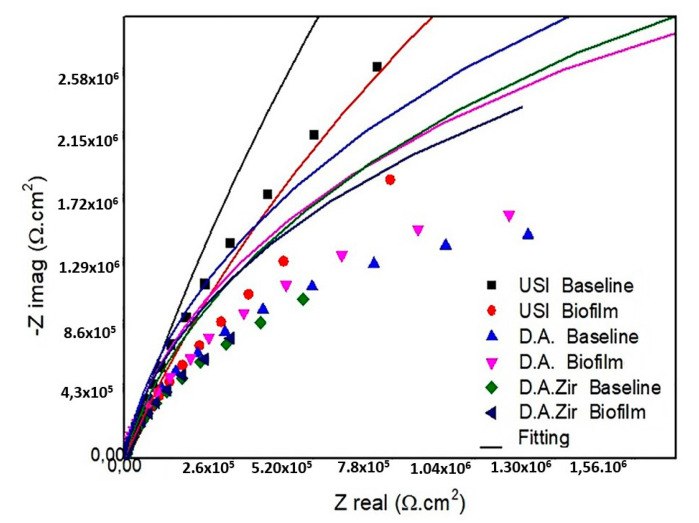
Diagram of the Nyquist values of the surfaces tested are as follows: USI (BHI and Biofilm), D.A. (BHI and Biofilm), and D.A.Zir (BHI and Biofilm).

The evolution of the OCP as a function of time is shown in Figure 3. Regarding the different surfaces in the intragroup analysis of the electrolyte (BHI), there was no statistical difference (*p* > 0.05) between the tests (D.A. and D.A.Zir) and control surfaces (USI). Similarly, in the intragroup analysis of the biofilm electrolyte, there was no statistically significant difference (*p* > 0.05) observed between the tests and control surfaces or between all the three surfaces that were evaluated. However, higher OCP values were recorded for the D.A. (BHI) group. In the analysis as a function of electrolyte, no statistical difference was observed between the groups (*p* = 0.3681). The D.A. in the BHI group presented the highest values for OCP, followed by the D.A.Zir in Biofilm, USI in Biofilm, USI in BHI, D.A.Zir in BHI, and D.A. in Biofilm groups. In the intra-group evaluation, all had *p*-values greater than 0.05.

#### 3.2.2. Electrochemical Impedance Spectroscopy

In the presentation of the EIS data (Figure 3), the Nyquist diagram (real component—|Z| real impedance versus imaginary component—|Zimg| impedance) shows that the USI (BHI) had the largest semicircular diameter of the capacitance loop, followed by USI (Biofilm) and DA (Biofilm).

**Figure 4:**
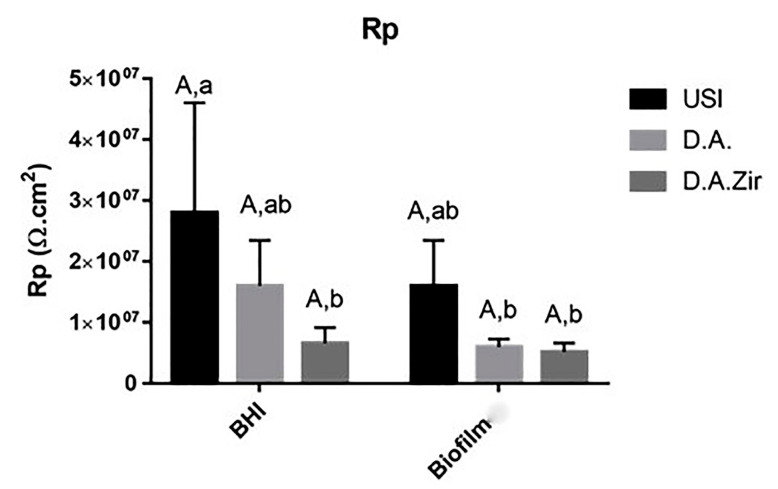
Graphical representation of the means and standard deviations of the Rp values for the USI, D.A. and D.A.Zir groups in Biofilm and BHI. Equal capital letters show statistical similarity between the surfaces. Equal lowercase letters show statistical similarity with respect to the electrolytes (BHI and Biofilm) between the groups.

In the BODE diagram (Figure 4), it can be observed that, at low frequencies, only the D.A.Zir (Biofilm) group presented the lowest value for the impedance modulus followed by the D.A.Zir (BHI) group. Conversely, the USI (BHI), D.A. (BHI), and USI (Biofilm) groups had higher values—indicating that they maintained high frequencies.

The Rp values, in relation to the different surfaces in the intragroup analysis of the BHI and biofilm electrolytes, as well as the interaction between these surfaces and between these factors, did not show statistical differences (*p* = 0.360) (Figure 3). At the 5% significance level for the Tukey test, considering only the electrolyte parameter (BHI and Biofilm), there was a statistical difference between USI BHI and D.A. Biofilm (*p* = 0.0058), USI BHI and D.A. Biofilm (*p* = 0.0041), and USI BHI and D.A.Zir BHI (*p* = 0.0077).

#### 3.2.3. Potentiodynamic Electrochemical Test

**Figure 5:**
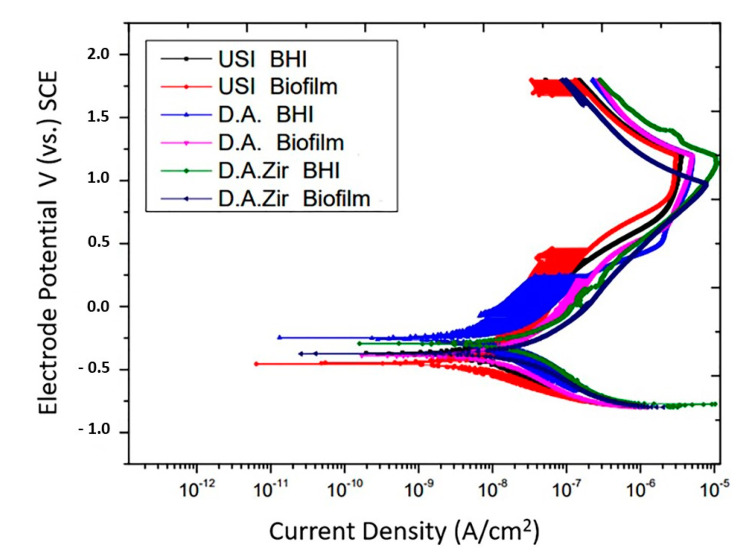
Potentiodynamic curves of the USI, D.A. and D.A.Zir groups as a function of electrolytes (BHI and Biofilm).

In the representation of the results through the potentiodynamic curve, all groups presented regions of active–passive transition (Figure 5).

Overall, all groups had similar passivation constants. In addition to presenting late passivation, this one did not present a constant. In fact, it presented a curve in the passivation region, which took longer to reach the passivity point.

The passivation current, in ascending order with respect to the most favorable electrochemical behavior, is USI (BHI) > USI (Biofilm) > O.D. (BHI) = O.D. (Biofilm) > O.D. Zir (BHI) > O.D. Zir (Biofilm).

**Figure 6:**
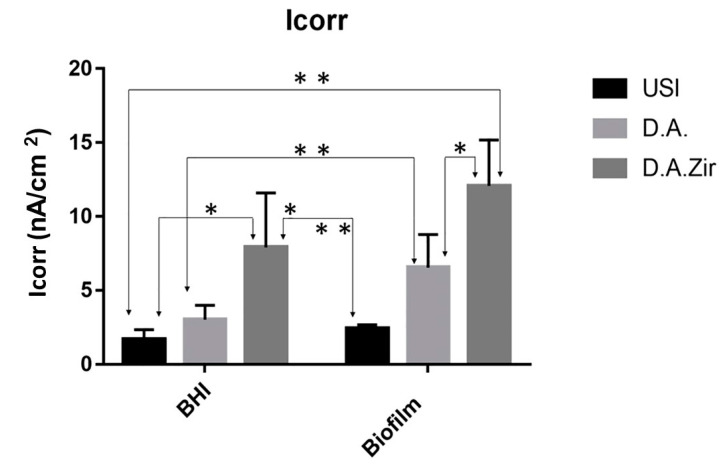
Graphical representation of the means and standard deviations of the Icorr values for the groups of surfaces (USI, D.A. and D.A.Zir as a function of electrolytes (BHI and Biofilm)). A single asterisk on the indication of the specimens corresponds to the electrolyte factor. Asterisks over the specimen indication correspond to the surface factor.

With respect to electrolytes and biofilms, there was no statistical difference in Icorr between the groups (*p* = 0.2868). For inter-group analysis considering the “electrolyte” factor, there was a difference between the groups (Figure 6).

### 3.3. Atomic Force Microscopy

**Figure 7:**
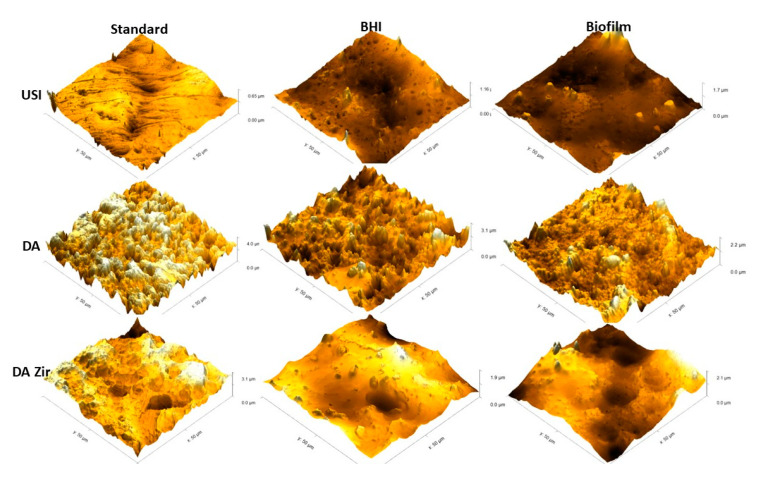
Images obtained by MFA in an area of 50 µm by 50 µm on the surface of the Machined (USI), Double Acid Etch (D.A.) and Double Acid Etch and Zirconia Blasting (D.A.Zir) discs. Representing [USI, D.A. and D.A.Zir (Standard)] (disc received by the manufacturer for testing); [USI, D.A. and D.A.Zir (BHI)] (disk that underwent the electrochemical test without biofilm growth for 7 days) and; [USI, D.A. and D.A.Zir (Biofilm)] (disk that underwent the microbiological test for biofilm growth for 7 days followed by the electrochemical test).

Figure 7 represents the 3D images.

In general, it is observed that the USI surface is more homogeneous than the others (D.A. and D.A.Zir)—regardless of the biofilm and electrochemical assay. However, it is observed that there is a greater difference in altitude from a surface of 0.65 µm to another of 1.7 µm after the corrosion testing in the presence of *E. faecalis*. However, the surface treated with D.A. has well-defined peaks and valleys, albeit with a decrease in the specimen’s altitude after the corrosion test and by contact with the biofilm (from 3.1 µm to 2.1 µm). This is due to deposition of organic and biological materials on the surface, making it less irregular and attenuating the altitude of peaks and valleys.

The D.A.ZIr group shows a greater amount of peaks and valleys compared to the other two surfaces. This is indicative of large surface irregularities—observed by a decrease in roughness (from 4.0 µm to 2.2 µm) after the corrosion test, and this can be attributed to deposition of the BHI broth compounds.

## 4. Discussion

From the microbiological results presented, it can be observed that surface modifications facilitated bacterial aggregation when compared to the machined surfaces (Figure 2). In the D.A.Zir group, a greater number of bacteria adhered, owing to its more irregular surface (Figure 7). Bacterial adhesion is easy on a rough surface, especially inside the surface irregularities where the valleys are located [37]. However, it ends up favoring the speed of osseointegration and the success rate [05]. Regarding the surface roughness that facilitates bacterial adhesion to implants (Figure 2), there has been an instance wherein the addition of antibacterial coatings was able to inhibit infections [38] just as it inhibits microbial adhesion. Studies have been conducted to improve cell–surface integration, especially with efforts in recent years to develop surfaces with antibacterial characteristics, such as non-stick surfaces, introduction of drugs or metal ions on surfaces, and inorganic agents (silver ions) [39,40,41]. There is a clinical practice of administering drugs to the body, often taken orally, to prevent bacterial infection by sticking onto the implanted material and thus preventing the formation of a bacterial biofilm on the surface [42].

Scientists have worked to improve the quality of implant surfaces for anti-inflammatory and antimicrobial events [43]. However, surface alteration methods are still being used. The subtraction method is quite common for the treatment of implant surfaces. The subtraction on the titanium surface is made with aluminum oxide blasting [44], bio-ceramic particles [45] of various sizes, or acid etching, giving a rough, micro-textured, biocompatible surface in which implants are treated with an ultrasonic bath, rinsed, and finally dried [46].

Corroborating this information, the surface modifications in this study by blasting with zirconia and acid etching had greater microbial adhesion (Figure 2), and this can be attributed to the optimization of surface roughness [47]. This is in agreement with the MFA images, which show irregularities of the modified surfaces pertaining to the machined surfaces (Figure 6), corroborating the quantitative data from the bacterial adhesion analysis (Figure 2).

The surface that was modified by sandblasting, followed by double acid etching (D.A.Zir), had the greatest surface irregularity (Figure 6—D.A.Zir [Standard]). This modified surface without the addition of zirconia particles (Figure 7—D.A.Zir [Standard]) is facilitated by acid etching, which removes residues [47].

It is believed that colony formation was facilitated by the large number of peaks and valleys present in the D.A.Zir group, and this was not observed in the USI group. This experimental observation suggests changes in the surface of dental implants, with texturization of the apical and middle thirds to favor osseointegration. In the cervical third and implant platform with a polished surface, as it is often above the bone, it would minimize microbial and attenuate the release of titanium particles to the oral surface by corrosion submitted to the implant surface [48].

In the D.A. group, there was no statistically significant difference observed at the 5% level compared to the USI and D.A.Zir groups, both for the microbiological assay. Notably, the surface modification of the A.A. group presented less irregularities than the D.A.Zir group (Figure 7), but more than that of the USI group (Figure 6). The difference in colonies between D.A.Zir and USI may have caused, in given episodes, a reduction in local pH, due to microbial colonization on the surfaces [49]. In the presence of low pH, the corrosive behavior of Ti is more pronounced.

This relationship between the presence of biofilm and a decrease in the ability to resist corrosion was found in this study (Figure 4), albeit with no statistical difference for some of the factors to be discussed. This is because these colonizers deal damage to the colonized surfaces [50].

It can be observed that the higher value of CFU/mL in the D.A.Zir group (BHI) resulted in a lower capacity to resist the corrosive process (Figure 4), especially when considering the polarization cycle that causes the disturbance of the biofilm that functions as a lubricant for corrosive events [51]; that is, the greatest irregularity was unable to resist corrosion. Furthermore, the corrosion of Ti increases its surface roughness [48], and this increases the area for electrochemical integration, further destabilizing its surface. This causes the release of Ti corrosion products into the environment, with peri-implant biological consequences, tissue accumulation [52], and with the presence of a foreign body, inflammation, pain, peri-implantitis, and loss of implants [10,11]. It is desirable that the application of Ti in the form of dental and orthopedic implants has a low corrosion rate [53]. Thus, hypothesis H0, that is, biofilm forms in greater quantity on the surface with double acid attack, was accepted.

The parameters to which the groups were submitted correspond to the OCP, EIS, and the potentiodynamic polarization curve. The electrochemical corrosion reactions on the surface of the specimens used in this study required a humid or electrochemical environment, which is free from mechanical corrosion where functional stresses on the implant could compromise it [54,55]. For OCP, the D.A.Zir (Biofilm) groups exhibited the best behavior for recovering the electrical potential in lesser time. Groups D.A. (BHI) and D.A.Zir (BHI) showed good stabilization of the system, at the beginning, to resist corrosion as well. OCP showed no statistical difference for the surface (*p* = 0.9891) and electrolyte (*p* = 0.3681), and this analysis indicates that the presence of the biofilm on the specimens influenced the electrochemical stabilization by causing the biofilm to protect against corrosive events. It should be noted that the OCP makes it possible to assess the potential of the material before the solution and stabilize the system.

For the Nyquist diagram, which is intended to provide greater resistance to charge transfer and lesser potential for ionic exchanges with the external environment, the larger semi-circular diameter of the USI (BHI) and USI (Biofilm) denotes good electrochemical behavior. This is also observed for the D.A. (Biofilm), but without major differences from the other D.A.Zir (BHI and Biofilm) and D.A. (BHI) groups. If there are fewer surface irregularities or pores, there may be lesser ionic exchanges with the medium [56,57]. Ti surfaces exposed to changes in the pH of the low oral environment, as is observed with the use of fluoridated toothpastes that promote acidification of the medium with damage to the TiO_2_ layer, become vulnerable to corrosion [58,59,60].

In oral rehabilitation, no ions should be released due to corrosion [55]. Even though it is the dissolution of Ti alloys, this can lead to toxicity [61]. In this sense, when analyzing the BODE diagram, it can be observed that the USI (BHI), D.A. (BHI), and USI (Biofilm) groups have a good capacity to resist ionic exchanges with the medium. The smaller surface irregularity provides a smaller ion exchange, as can be observed for the USI (BHI) and D.A. (BHI) groups. Conversely, D.A.Zir (Biofilm) had greater ion exchange. In this case, the machined surface provided better electrochemical stability, and the presence of the biofilm did not protect the surface in a corrosive event. As noted, the USI group’s lower ionic exchange (BHI) results in a lower corrosive process that does not disturb the oxide layer [55]. For the dynamic polarization potential, the D.A.Zir (BHI) and D.A.Zir (Biofilm) displayed adverse electrochemical behavior. For the corrosion current density (Icorr), when compared the surfaces of the groups and of these of the electrolytes, there was a statistically significant difference. A better ability to resist generalized corrosion was observed in the USI group (BHI and Biofilm). The analysis of Figure 6 allows us to observe a greater corrosion active region and a longer delay reaching the passive region for D.A.Zir (BHI) and D.A.Zir (Biofilm). In the case of colonization by *Porphyromonas gingivalis* for 30 days in implants, corrosion of the implants was induced, as well as the presence of severe attacks of corrosion, by surface discoloration and increased surface roughness [62].

Knowing the dimensions of the valleys, pores, and cracks on the surface in relation SRIDHAR n to the size of the bacteria is important in terms of fixation, and even more so in the presence of artificial saliva that contains mucin protein that favors surface adhesion [63]. The analysis of Figure 6 shows that specimens that have gone through a period for biofilm formation, compared to those immersed in BHI, have higher corrosion current density values. That is, they have poor electrochemical behavior, as harmful products from bacteria can dissolve the titanium oxide layer, initiating the corrosive cycle [64].

Given the other hypotheses that were raised pertaining to the electrochemical tests, with the presence of biofilm to protect the Ti surface with greater passivation against electric current, it was observed that the presence of the biofilm resulted in a more diminished capacity of the specimens to resist ionic exchanges with the medium. This statement is confirmed by the results presented by Rp (Figure 4) and Icorr (Figure 6).

Biofilm brings severe metabolic activity of corrosive metal substances to the environment [19,65], that is, in another situation, the surface degradation with the dissolution of metallic particles to the in vivo [65].

The is different from the adhesion of bacteria as it can serve as a more preventive method for the dissolution of metal ions in the electrolyte [50,65]. There was worse electrochemical behavior for DAZir (Biofilm) When compared to th Other groups, as shown by the BODE, Nyquist and Icorr diagrams. However, the USI group showed better behavior for ionic exchange. It can be observed that the greater the surface irregularity, favored the formation of more biofilm, which consequently led to greater ionic exchanges with the medium.

These findings allow us to affirm that the biofilm did not decrease the resistance of Ti to corrosion (H1 rejects), as D.A.Zir had greater microbial adhesion capacity and greater ion exchange capacity with the medium.

## 5. Conclusions

Given the results, the limitations of the study, and suggestions in relation to corrosion on surfaces due to the biofilm in the evaluated time, the conclusions are as follows:

- There was greater formation of biofilm on the Ti surface with zirconia blasting, followed by double acid etching (D.A.Zir).
- The presence of biofilm influenced the increase in corrosion.
- The D.A.Zir (Biofilm) group exhibited the most corrosive behavior.

Further studies considering the interaction of the biofilm and the tribocorrosion on titanium with surface treatment are necessary in order to investigate a surface with less ion exchange when in contact with the biofilm.

## Data Availability

Not applicable.

## References

[1] Hatamleh M.M., Wu X., Alnazzawi A., Watson J., Watts D. (2018). Surface characteristics and biocompatibility of cranioplasty titanium implants following different surface treatments. Dent. Mater..

[2] Sarraf M., Rezvani Ghomi E., Alipour S., Ramakrishna S., Liana Sukiman N. (2022). A state-of-the-art review of the fabrication and characteristics of titanium and its alloys for biomedical applications. Biodes. Manuf..

[3] Szczęsny G., Kopec M., Politis D.J., Kowalewski Z.L., Łazarski A., Szolc T. (2022). A Review on Biomaterials for Orthopaedic Surgery and Traumatology: From Past to Present. Materials.

[4] Kligman S., Ren Z., Chung C.H., Perillo M.A., Chang Y.C., Koo H., Zheng Z., Li C. (2021). The Impact of Dental Implant Surface Modifications on Osseointegration and Biofilm Formation. J. Clin. Med..

[5] Petrini M., Giuliani A., Di Campli E., Di Lodovico S., Iezzi G., Piattelli A., D’Ercole S. (2020). The Bacterial Anti-Adhesive Activity of Double-Etched Titanium (DAE) as a Dental Implant Surface. Int. J. Mol. Sci..

[6] Queiroz T.P., Souza F.Á., Guastaldi A.C., Margonar R., Garcia-Júnior I.R., Hochuli-Vieira E. (2013). Commercially pure titanium implants with surfaces modified by laser beam with and without chemical deposition of apatite. Biomechanical and topographical analysis in rabbits. Clin. Oral Implants Res..

[7] Schwarz F., Alcoforado G., Guerrero A., Jönsson D., Klinge B., Lang N., Mattheos N., Mertens B., Pitta J., Ramanauskaite A. (2021). Peri-implantitis: Summary and consensus statements of group 3. The 6th EAO Consensus Conference 2021. Clin. Oral Implants Res..

[8] Stavropoulos A., Bertl K., Winning L., Polyzois I. (2021). What is the influence of implant surface characteristics and/or implant material on the incidence and progression of peri-implantitis? A systematic literature review. Clin. Oral Implants Res..

[9] Zhao B., van der Mei H.C., Subbiahdoss G., de Vries J., Rustema-Abbing M., Kuijer R., Busscher H.J., Ren Y. (2014). Soft tissue integration versus early biofilm formation on different dental implant materials. Dent. Mater..

[10] De Melo F., do Nascimento C., Souza D.O., de Albuquerque R.F. (2017). Identification of oral bacteria on titanium implant surfaces by 16S rDNA sequencing. Clin. Oral Implants Res..

[11] Ferreira Ribeiro C., Cogo-Müller K., Franco G.C., Silva-Concílio L.R., Sampaio Campos M., de Mello Rode S., Claro Neves A.C. (2016). Initial oral biofilm formation on titanium implants with different surface treatments: An in vivo study. Arch. Oral Biol..

[12] Guglielmotti M.B., Olmedo D.G., Cabrini R.L. (2019). Research on implants and osseointegration. Periodontol. 2000.

[13] Hallab N., Merritt K., Jacobs J.J. (2001). Metal sensitivity in patients with orthopaedic implants. J. Bone Joint Surg. Am..

[14] Gonçalves I.M.R., Herrero E.R., Carvalho O., Henriques B., Silva F.S., Teughels W., Souza J.C.M. (2021). Antibiofilm effects of titanium surfaces modified by laser texturing and hot-pressing sintering with silver. J. Biomed. Mater. Res. B Appl. Biomater..

[15] Van Hengel I.A.J., Putra N.E., Tierolf M.W.A.M., Minneboo M., Fluit A.C., Fratila-Apachitei L.E., Apachitei I., Zadpoor A.A. (2020). Biofunctionalization of selective laser melted porous titanium using silver and zinc nanoparticles to prevent infections by antibiotic-resistant bacteria. Acta Biomater..

[16] D’Ercole S., Cellini L., Pilato S., Di Lodovico S., Iezzi G., Piattelli A., Petrini M. (2020). Material characterization and Streptococcus oralis adhesion on Polyetheretherketone (PEEK) and titanium surfaces used in implantology. J. Mater. Sci. Mater. Med..

[17] Schwarz F., Derks J., Monje A., Wang H.L. (2018). Peri-implantitis. J. Clin. Periodontol..

[18] Videla H.A., Herrera L.K. (2005). Microbiologically influenced corrosion: Looking to the future. Int. Microbiol..

[19] Fukushima A., Mayanagi G., Nakajo K., Sasaki K., Takahashi N. (2014). Microbiologically induced corrosive properties of the titanium surface. J. Dent. Res..

[20] Wang X., Ning B., Pei X. (2021). Tantalum and its derivatives in orthopedic and dental implants: Osteogenesis and antibacterial properties. Colloids Surf. B Biointerfaces.

[21] Koike M., Fujii H. (2001). The corrosion resistance of pure titanium in organic acids. Biomaterials.

[22] Bearinger J.P., Orme C.A., Gilbert J.L. (2003). Effect of hydrogen peroxide on titanium surfaces: In situ imaging and step-polarization impedance spectroscopy of commercially pure titanium and titanium, 6-aluminum, 4-vanadium. J. Biomed. Mater. Res. A.

[23] Fabricius L., Dahlén G., Holm S.E., Möller A.J. (1982). Influence of combinations of oral bacteria on periapical tissues of monkeys. Scand. J. Dent. Res..

[24] Farrow J.A., Jones D., Phillips B.A., Collins M.D. (1983). Taxonomic studies on some group D streptococci. J. Gen. Microbiol..

[25] Bystrom A., Claesson R., Sundqvist G. (1985). The antibacterial effect of camphorated paramonochlorophenol, camphorated phenol and calcium hydroxide in the treatment of infected root canals. Endod. Dent. Traumatol..

[26] Graham C.E., Cruz M.R., Garsin D.A., Lorenz M.C. (2017). *Enterococcus faecalis* bacteriocin EntV inhibits hyphal morphogenesis, biofilm formation, and virulence of *Candida albicans*. Proc. Natl. Acad. Sci. USA.

[27] Ji Y., Han Z., Ding H., Xu X., Wang D., Zhu Y., An F., Tang S., Zhang H., Deng J. (2021). Enhanced Eradication of Bacterial/Fungi Biofilms by Glucose Oxidase-Modified Magnetic Nanoparticles as a Potential Treatment for Persistent Endodontic Infections. ACS Appl. Mater. Interfaces.

[28] Rôças I.N., Siqueira J.F., Santos K.R. (2004). Association of Enterococcus faecalis with different forms of periradicular diseases. J. Endod..

[29] Heidari H., Hasanpour S., Ebrahim-Saraie H.S., Motamedifar M. (2017). High Incidence of Virulence Factors Among Clinical Enterococcus faecalis Isolates in Southwestern Iran. Infect. Chemother..

[30] Faverani L.P., Barao V.A., Pires M.F., Yuan J.C., Sukotjo C., Mathew M.T., Assunção W.G. (2014). Corrosion kinetics and topography analysis of Ti-6Al-4V alloy subjected to different mouthwash solutions. Mater. Sci. Eng. C Mater. Biol. Appl..

[31] Barão V.A., Mathew M.T., Assunção W.G., Yuan J.C., Wimmer M.A., Sukotjo C. (2011). The role of lipopolysaccharide on the electrochemical behavior of titanium. J. Dent. Res..

[32] Gao Y., Jiang X., Lin D., Chen Y., Tong Z. (2016). The Starvation Resistance and Biofilm Formation of Enterococcus faecalis in Coexistence with Candida albicans, Streptococcus gordonii, Actinomyces viscosus, or Lactobacillus acidophilus. J. Endod..

[33] Andreotti A.M., Sousa C.A., Goiato M.C., Silva E.V.F.D., Duque C., Moreno A., Santoso D.M.D. (2018). In vitro evaluation of microbial adhesion on the different surface roughness of acrylic resin specific for ocular prosthesis. Eur. J. Dent..

[34] Barão V.A., Mathew M.T., Assunção W.G., Yuan J.C., Wimmer M.A., Sukotjo C. (2012). Stability of cp-Ti and Ti-6Al-4V alloy for dental implants as a function of saliva pH—An electrochemical study. Clin. Oral Implants Res..

[35] Canty M.K., Hansen L.A., Tobias M., Spencer S., Henry T., Luke-Marshall N.R., Campagnari A.A., Ehrensberger M.T. (2019). Antibiotics Enhance Prevention and Eradication Efficacy of Cathodic-Voltage-Controlled Electrical Stimulation against Titanium-Associated Methicillin-Resistant Staphylococcus aureus and Pseudomonas aeruginosa Biofilms. mSphere.

[36] Canty M., Luke-Marshall N., Campagnari A., Ehrensberger M. (2017). Cathodic voltage-controlled electrical stimulation of titanium for prevention of methicillin-resistant Staphylococcus aureus and Acinetobacter baumannii biofilm infections. Acta Biomater..

[37] Ribeiro A.V., Velásquez-Espedilla E.G., de Barros M.C., de Melo Simas L.L., de Andrade F.B. (2023). Influence of Gutta-Percha Surface on *Enterococcus faecalis* Initial Adhesion In Vitro: An Atomic Force Microscopy Study. Life.

[38] Patelli A., Mussano F., Brun P., Genova T., Ambrosi E., Michieli N., Mattei G., Scopece P., Moroni L. (2018). Nanoroughness, Surface Chemistry, and Drug Delivery Control by Atmospheric Plasma Jet on Implantable Devices. ACS Appl. Mater. Interfaces.

[39] Rai M., Yadav A., Gade A. (2009). Silver nanoparticles as a new generation of antimicrobials. Biotechnol. Adv..

[40] Karcı B., Öncü E., Dogan M. (2021). The Effect of Different Dental Implant Surface Characteristics on Bone Immunologic Biomarkers and Microbiologic Parameters: A Randomized Clinical Study. Int J Periodontics Restor. Dent..

[41] De Vos M.G.J., Zagorski M., McNally A., Bollenbach T. (2017). Interaction networks, ecological stability, and collective antibiotic tolerance in polymicrobial infections. Proc. Natl. Acad. Sci. USA.

[42] Ferraris S., Cochis A., Cazzola M., Tortello M., Scalia A., Spriano S., Rimondini L. (2019). Cytocompatible and Anti-bacterial Adhesion Nanotextured Titanium Oxide Layer on Titanium Surfaces for Dental and Orthopedic Implants. Front. Bioeng. Biotechnol..

[43] Li X., Qi M., Sun X., Weir M.D., Tay F.R., Oates T.W., Dong B., Zhou Y., Wang L., Xu H.H.K. (2019). Surface treatments on titanium implants via nanostructured ceria for antibacterial and anti-inflammatory capabilities. Acta Biomater..

[44] Chen C.J., Ding S.J., Chen C.C. (2016). Effects of Surface Conditions of Titanium Dental Implants on Bacterial Adhesion. Photomed. Laser Surg..

[45] Müeller W.D., Gross U., Fritz T., Voigt C., Fischer P., Berger G., Rogaschewski S., Lange K.P. (2003). Evaluation of the interface between bone and titanium surfaces being blasted by aluminium oxide or bioceramic particles. Clin. Oral Implants Res..

[46] Buser D., Schenk R.K., Steinemann S., Fiorellini J.P., Fox C.H., Stich H. (1991). Influence of surface characteristics on bone integration of titanium implants. A histomorphometric study in miniature pigs. J. Biomed. Mater. Res..

[47] Karthigeyan S., Ravindran A.J., Bhat R.T.R., Nageshwarao M.N., Murugesan S.V., Angamuthu V. (2019). Surface Modification Techniques for Zirconia-Based Bioceramics: A Review. J. Pharm. Bioallied Sci..

[48] Mombelli A., Hashim D., Cionca N. (2018). What is the impact of titanium particles and biocorrosion on implant survival and complications? A critical review. Clin. Oral Implants Res..

[49] Zhang Y., Gulati K., Li Z., Di P., Liu Y. (2021). Dental Implant Nano-Engineering: Advances, Limitations and Future Directions. Nanomaterials.

[50] Sridhar S., Wang F., Wilson TGJr Valderrama P., Palmer K., Rodrigues D.C. (2018). Multifaceted roles of environmental factors toward dental implant performance: Observations from clinical retrievals and in vitro testing. Dent. Mater..

[51] Dini C.R., Costa R., Sukojito C., Takoudis C.G., Mateus M.T., Barão V. (2020). Progression of bio-tribocorrosion in implant dentistry. Front. Mech. Eng..

[52] Li T., Gulati K., Wang N., Zhang Z., Ivanovski S. (2018). Preenchendo a lacuna: Fabricação otimizada de nanoestruturas robustas de titânia em geometrias complexas de implantes para tradução clínica. J. Colloid Interface Sci..

[53] Zhang E., Liu C. (2015). Effect of surface treatments on the surface morphology, corrosion property, and antibacterial property of Ti-10Cu sintered alloy. Biomed. Mater..

[54] Tschernitschek H., Borchers L., Geurtsen W. (2005). Nonalloyed titanium as a bioinert metal—A review. Quintessence Int..

[55] Noumbissi S., Scarano A., Gupta S. (2019). A Literature Review Study on Atomic Ions Dissolution of Titanium and Its Alloys in Implant Dentistry. Materials.

[56] Faverani L.P., Barão V.A., Ramalho-Ferreira G., Ferreira M.B., Garcia-Júnior I.R., Assunção W.G. (2014). Effect of bleaching agents and soft drink on titanium surface topography. J. Biomed. Mater. Res. B Appl. Biomater..

[57] Beline T., Garcia C.S., Ogawa E.S., Marques I.S.V., Matos A.O., Sukotjo C., Mathew M.T., Mesquita M.F., Consani R.X., Barão V.A.R. (2016). Surface treatment influences electrochemical stability of cpTi exposed to mouthwashes. Mater. Sci. Eng. C Mater. Biol. Appl..

[58] Siirilä H.S., Könönen M. (1991). The effect of oral topical fluorides on the surface of commercially pure titanium. Int. J. Oral Maxillofac. Implants.

[59] Lugowski S.J., Smith D.C., McHugh A.D., Van Loon J.C. (1991). Release of metal ions from dental implant materials in vivo: Determination of Al, Co, Cr, Mo, Ni, V, and Ti in organ tissue. J. Biomed. Mater. Res..

[60] Anwar E.M., Kheiralla L.S., Tammam R.H. (2011). Effect of fluoride on the corrosion behavior of Ti and Ti6Al4V dental implants coupled with different superstructures. J. Oral Implantol..

[61] Sedarat C., Harmand M.F., Naji A., Nowzari H. (2001). In vitro kinetic evaluation of titanium alloy biodegradation. J. Periodontal Res..

[62] Rodrigues D.C., Valderrama P., Wilson T.G., Palmer K., Thomas A., Sridhar S., Adapalli A., Burbano M., Wadhwani C. (2013). Titanium Corrosion Mechanisms in the Oral Environment: A Retrieval Study. Materials.

[63] Narendrakumar K., Kulkarni M., Addison O., Mazare A., Junkar I., Schmuki P., Sammons R., Iglič A. (2015). Adherence of oral streptococci to nanostructured titanium surfaces. Dent. Mater..

[64] Delgado-Ruiz R., Romanos G. (2018). Potential Causes of Titanium Particle and Ion Release in Implant Dentistry: A Systematic Review. Int. J. Mol. Sci..

[65] Sridhar S., Wang F., Wilson T.G., Palmer K., Valderrama P., Rodrigues D.C. (2019). The role of bacterial biofilm and mechanical forces in modulating dental implant failures. J. Mech. Behav. Biomed. Mater..

